# Dechlorination of Hexachlorobenzene in Contaminated Soils Using a Nanometallic Al/CaO Dispersion Mixture: Optimization through Response Surface Methodology

**DOI:** 10.3390/ijerph15050872

**Published:** 2018-04-27

**Authors:** Yuhui Jiang, Yixuan Shang, Shuyao Yu, Jianguo Liu

**Affiliations:** Key Laboratory for Solid Waste Management and Environment Safety (Tsinghua University), Ministry of Education of China, Tsinghua University, Beijing 100084, China; jiangyh13@mails.tsinghua.edu.cn (Y.J.); shang14@mails.tsinghua.edu.cn (Y.S.); yujadesj@126.com (S.Y.)

**Keywords:** nanometallic Al/CaO, ball milling, dechlorination, response surface methodology

## Abstract

Hexachlorobenzene (HCB) contamination of soils remains a significant environmental challenge all over the world. Reductive stabilization is a developing technology that can decompose the HCB with a dechlorination process. A nanometallic Al/CaO (n-Al/CaO) dispersion mixture was developed utilizing ball-milling technology in this study. The dechlorination efficiency of HCB in contaminated soils by the n-Al/CaO grinding treatment was evaluated. Response surface methodology (RSM) was employed to investigate the effects of three variables (soil moisture content, n-Al/CaO dosage and grinding time) and the interactions between these variables under the Box-Behnken Design (BBD). A high regression coefficient value (*R*^2^ = 0.9807) and low *p* value (<0.0001) of the quadratic model indicated that the model was accurate in predicting the experimental results. The optimal soil moisture content, n-Al/CaO dosage, and grinding time were found to be 7% (m/m), 17.7% (m/m), and 24 h, respectively, in the experimental ranges and levels. Under optimal conditions, the dechlorination efficiency was 80%. The intermediate product analysis indicated that dechlorination was the process by stepwise loss of chloride atoms. The main pathway observed within 24 h was HCB → pentachlorobenzene (PeCB) → 1,2,3,4-tetrachlorobenzene (TeCB) and 1,2,4,5-TeCB. The results indicated that the moderate soil moisture content was crucial for the hydrodechlorination of HCB. A probable mechanism was proposed wherein water acted like a hydrogen donor and promoted the hydrodechlorination process. The potential application of n-Al/CaO is an environmentally-friendly and cost-effective option for decontamination of HCB-contaminated soils.

## 1. Introduction

In the past decades, contamination of subsurface soils by chlorinated hydrophobic organic compounds (CHOCs) such as hexachlorobenzene (HCB), polychlorinated biphenyls (PCBs), and polycyclic aromatic hydrocarbons (PAHs) has become a significant environmental problem [1]. Due to leakage and release from various industries, CHOCs present significant threats to the environment and human health. Soils are generally regarded as the final acceptor for the majority of CHOCs released to the environment [2]. Of all the soils contaminated by CHOCs, HCB-contaminated soils attract special environmental concern [3].

HCB was manufactured commercially in 1933 and has been extensively used as a fungicide and intermediate in organic chemical processes since then [4]. Due to its carcinogenicity, potential toxicity, and long-term persistence in the environment [5,6]. HCB contamination of soils presently poses a serious problem all over the world, including the USA, Japan [7], Germany [8], Australia [9], Brazil [10], and China. In 2001, HCB was classified as one of 12 ‘persistent organic pollutants’ (POPs) by the United Nations Environmental Program (UNEP). Despite the prohibition on HCB production across the world, it is still being generated unintentionally as a by-product in several industrial chemical processes [9,11]. In China, surveys suggest that there are 60~70 tons of HCB waste in stockpiles and at least six HCB-contaminated sites with large amounts of contaminated soils [12]. Remediation of HCB-contaminated soils remains a great challenge. Therefore, it is necessary to develop effective methods for the treatment of these soils.

Four major remediation technologies were used in order to remove organic pesticides and herbicides (HCB is one of them) from contaminated sites in the USA, including incineration, bioremediation, solidification/stabilization, and thermal desorption [13]. In recent years, various biologic, physical and chemical techniques have been studied for decontaminating HCB or other organics in these soils, including soil washing [14], electrokinetics [15], base-catalyzed dechlorination [16], nano zero-valent iron (nZVI) treatment [17], self-propagated sintering process [18], advanced oxidation process [19], and a biochar-plant tandem approach [20]. Although some of these technologies have demonstrated high HCB removal efficiencies, their application in practical remediation approaches is still a challenge.

The technology that can dechlorinate HCB is more promising, because HCB is greatly detoxified with the release of chlorine atoms [21]. Some studies have focused on the mechanochemical destruction of HCB by ball-milling. For example, the dechlorination efficiency by mechanochemical treatment with Fe/SiO_2_ was 100% after 6h ball-milling [22]. Similar observations were also found by several other researchers. The co-milling regents included CaH_2_ [23], CaO [24], Mg/Al_2_O_3_ [25], and CaC_2_ [26]. However, ball-milling cannot be used in large scale remediation of HCB-contaminated soils because of its high energy consumption. Therefore, some studies used ball-milling as a synthesis method to prepare a reductive material called nanometallic Ca/CaO dispersion mixture (n-Ca/CaO). This remediation technology was a simple grinding process (not ball-milling) of contaminated soils and n-Ca/CaO. After simple grinding with soils, n-Ca/CaO achieved 80% or more dechlorination efficiency of PCBs, polychlorinated dibenzodioxins (PCDDs), and polychlorinated dibenzofurans (PCDFs) [27,28]. However, n-Ca/CaO is too active to be stored in natural conditions (it must be stored in an Ar atmosphere) which limits the usage range and increases the overall cost. Additionally, this kind of material has not been used in HCB-contaminated soils. Furthermore, the effects of some important operating parameters such as soil moisture content, addition dosage, and grinding time on the dechlorination efficiency are not well understood.

In the present study, a cheaper reducing metal Al was inspired to replace Ca. The metal Al is less active, which may be stored in natural conditions. Based on the available literature, this is the first time Al has been used instead of Ca to develop a nanometallic aluminum/calcium oxide dispersion mixture (n-Al/CaO) by a mechanochemical technique. The dechlorination efficiency of HCB in soils by simple grinding with n-Al/CaO was evaluated. Response Surface Methodology (RSM) was employed for optimization of hexachlorobenzene dechlorination efficiency. The application variables (i.e., soil moisture, n-Al/CaO dosage and grinding time) were selected and optimized. A dechlorination pathway was identified by gas chromatography-mass spectrometer (GC-MS) and purge-trap gas chromatography-mass spectrometer (PT-GCMS), and the dechlorination mechanism of HCB was discussed.

## 2. Materials and Methods

### 2.1. Chemicals and Soils

HCB (99.0%), granular particles of metallic Al (99%, particle size distribution 0.5–2 μm), and CaO (98%, particle size distribution 1–5 μm) were purchased from Aladdin Chemicals, Shanghai, China. Natural soils collected from Jiangsu Huaian, China were air-dried, homogenized, and kept in an oven overnight at 105 °C for drying. Then the soils were ground and passed through a No. 50 (0.355 mm) sieve [29].

Fifty grams of the uncontaminated soils were spiked with 0.75 mL HCB acetone solution (1000 mg/L) and 20 mL pure acetone [30]. The simulated HCB contaminated soils were then dried in air for 24 h to evaporate acetone and stored in dark bottles prior to use. The theoretical HCB concentration was determined to be 15 mg/kg.

### 2.2. Preparation of n-Al/CaO

A dispersed mixture of Al and CaO was prepared through a planetary ball milling process. Granular particles of metallic Al and dried CaO (previously dried at 850 °C for 2 h) were introduced into the planetary ball mill (QM-3SP2; 200 g of stainless steel balls; diameter 1.2–30 mm) at a weight ratio (Al:CaO) of 1:2. The Al and CaO were milled for 1h at room temperature (25 °C) in an Ar gas atmosphere at 500 r/min. After milling, samples were collected and stored in a polyethylene hermetic bag.

### 2.3. Characterization of n-Al/CaO and Soils

The morphology and microstructure of n-Al/CaO was analyzed by a JSM-7001F scanning electron microscope (SEM, JEOL, Tokyo, Japan). X-ray diffraction (XRD) analysis was conducted using a Rigaku X-ray diffractometer (D/max 2500, Cu Kα radiation (1.5418 Å), and the accelerating voltage was 40 kV) (Rigaku, Tokyo, Japan). The n-Al/CaO particle size distribution was analyzed by the method of Dynamic Light Scattering (DelsaNano C, Beckman, Brea, CA, USA). The major and trace elemental composition of the soils was determined using a wavelength dispersive X-ray fluorescence spectrometer (XRF) equipped with a Rh X-ray tube and 4 kW generator (ARL PERFORM’X, Thermo Scientific, Waltham, MA, USA).

### 2.4. Treatment of Contaminated Soil with n-Al/CaO

The n-Al/CaO treatment was conducted in a cylindrical stainless-steel mixer (Φ 50 mm × 50 mm) with a stirring rake. Forty grams of soil samples were ground with different n-Al/CaO dosages in the mixer at a stirring speed of 120 r/min under room temperature (25 °C). Three variables including soil moisture content, n-Al/CaO dosage, and grinding (mixing) time were selected. The moisture content of the soils was adjusted by adding deionized water. All the experimental ranges and levels of these variables were determined by RSM in 2.7.

### 2.5. Analysis for Organic Compounds

For the quantitative analysis of semi-volatile organic compounds (SVOCs), two grams of soil samples after n-Al/CaO treatment and 100 nanograms of chlorobenzene substitutes (TCMX) were added into a cartridge. Then the SVOCs were extracted from the mixture with 300 mL of dichloromethane for 24 h. Before instrumental analysis, an injection standard (C_14_D_10_) was added. The extract was analyzed using a GC/MS (Thermo Fisher Trace ISQ, Waltham, MA, USA), and the internal standard method was used. The column used was DB-5MS (30 m × 0.25 mm × 0.25 μm). Helium (≥99.999%) at a constant flow rate (1.0 mL/min) was used as carrier gas. The GC-oven program was as follows: 80 °C held for 4 min, with a linear temperature gradient of 8 °C/min to 140 °C, and held for 3 min, with a linear temperature gradient of 3 °C/min to 160 °C, and a linear temperature gradient of 15 °C/min to 250 °C, held for 1 min. The injector temperature used was 250 °C and the injection volume was 1 μL. All of the analyses were completed using duplicate soil samples.

The adding standard recovery of the samples is 92–106%. The results proved to be reliable by adding standard recovery experiment in this process.

For the quantitative analysis of volatile organic compounds (VOCs), two grams of soil samples after n-Al/CaO treatment were collected to be analyzed with a PT-GCMS (Atomx—Thermo Fisher Trace ISQ). The VOCs were separated by GC and detected by MS. The GC-oven program was as follows: 35 °C held for 5 min, with a linear temperature gradient of 6 °C/min to 160 °C, and held for 5 min. The injector temperature was 220 °C. All of the analyses were completed using duplicate soil samples.

### 2.6. The Definition of HCB Dechlorination Efficiency

Due to the mass loss (~15%) of HCB during the simple grinding process, the total concentration (*C_T_*) of all the chlorinated benzenes was defined as follows to ensure comparability among the experimental results: [31]

$$C_{T}=\overset{n}{\underset{i=1}{\sum }}C_{i}$$

where *C_i_* is the concentration of the HCB and dechlorination products (e.g., HCB, PeCB, 1,2,3,4-TeCB, 1,2,4,5-TeCB and so on), and *n* is the number of all the chlorinated compounds detected with the analytical methods.

The hexachlorobenzene dechlorination efficiency (HDE) is defined as follows:

$$HDE=\left(1-\frac{C_{HCB}}{C_{T}}\right)\times 100\%$$

where *C_HCB_* is the concentration of HCB after n-Al/CaO treatment.

### 2.7. Experimental Design of RSM

RSM is generally employed to optimize the conditions of hexachlorobenzene dechlorination efficiency (HDE) by performing fewer experiments. For this study, the Box-Behnken design (BBD) was chosen to design the experiments in the RSM. The BBD is well suited for process optimization of several variables [32]. The optimum condition of the variables considered in this study including soil moisture content (A), n-Al/CaO dosage (B), and grinding time (C) were evaluated. The HDE was taken as the response function for this optimization study. The low, middle, and high level of the variables were coded as −1, 0 and +1 as shown in Table 1. The range and level of each variable was chosen with economic and operational considerations. For example, excess n-Al/CaO dosage may lead to the soil component changes, and more undesirable operational costs. Therefore, a total of 17 experiments were designed by Design Expert (version 8.0, Stat-Ease, Minneapolis, MN, USA) and carried out randomly (Table 2). Analysis of variance (ANOVA) was employed to evaluate the validity of the predictive model. The regression coefficient value (*R*^2^) was used to evaluate the effectiveness of the predictive model.

## 3. Results and Discussion

### 3.1. Characterization of Contaminated Soils and n-Al/CaO

The soils were silica rich and contained up to 78.1% SiO_2_, 9.8% Al_2_O_3_, 9.1% CaO, 2.9% Fe_2_O_3_, and less than 0.1% of K_2_O, Na_2_O, and MgO. Other primary physical and chemical characteristics of soils were as follows: organic matter content of 48.6 g/kg (LY/T 1237–1999), pH of 9.1 (NY/T 1377–2007), electrical conductivity of 67.9 μs/cm (HJ 802–2016), cation exchange capacity of 2.01 cmol/kg (NY/T 1121.5–2006), and oxidation-reduction potential of 444 mV (HJ 746–2015). The determined HCB concentration of contaminated soils was 12 mg/kg.

**Figure 1:**
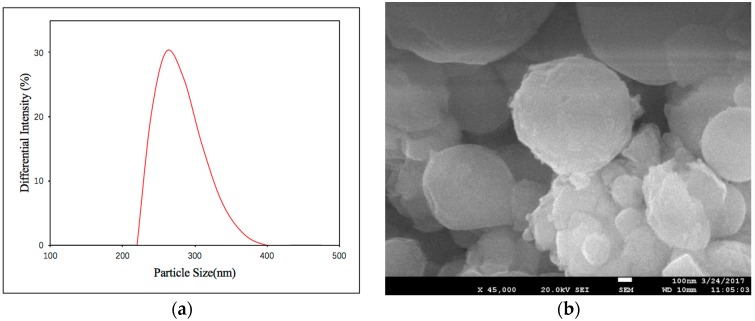
Nanometallic Al/CaO: (**a**) Particle size distribution; (**b**) SEM microstructure.

**Figure 2:**
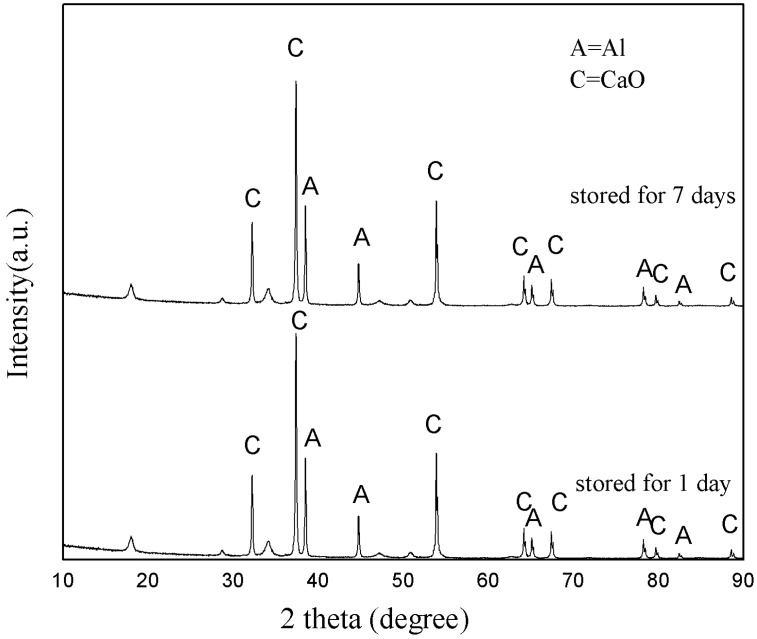
XRD patterns of n-Al/CaO stored for one day and seven days.

The particle size distribution and SEM microstructure are presented in Figure 1. The average size of the n-Al/CaO particles was 277 nm (Figure 1a). Through XRD analysis, regardless of the storage time of one day or seven days, the diffraction peaks confirmed the presence of Al and CaO in the n-Al/CaO sample (Figure 2). These results indicated acceptable stability under long-term storage conditions for the compositions.

### 3.2. Experimental Results and ANOVA Analysis

Three selected variables were optimized through RSM analysis under BBD to determine the maximum dechlorination efficiency of HCB. The design experiments and experimental responses are presented in Table 2. The analysis of variance (ANOVA) is shown in Table 3 and a predictive quadratic equation that incorporates the interactions between the three variables is described as follows:

$$\mathrm{HDE}=-16.390+10.572A+3.335B+2.784C+0.209AB+0.138AC+0.152BC-1.229A^{2}-0.221B^{2}-0.155C^{2}$$

where HDE is the dechlorination efficiency of HCB. *A*, *B* and *C* represent the soil moisture content, n-Al/CaO dosage, and grinding time, respectively.

Table 3 shows the ANOVA of the predictive model. The very low values of Prob > F (<0.0001) indicated the model was significant. Moreover, based on the values Prob > F (<0.05), all three variables were found to be significant model terms. The results indicated that soil moisture content (A), n-Al/CaO dosage (B) and grinding time (C) in the response could be explained by the predictive quadratic model and had prominent effects on the dechlorination of HCB. The lack of fit Prob > F value (0.0553) of the model showed that the variation of the data around the predictive quadratic model was not significant relative to the pure error, implying significant model correlation between the three variables and the response. The ANOVA results showed that the model successfully described the correlation between the variables and the response (HDE). Furthermore, the regression coefficient of the model was high (*R*^2^ = 0.9807), which implied that more than 98% of the variations for HCB dechlorination efficiency could be explained by the three variables (soil moisture content, n-Al/CaO dosage, and grinding time).

### 3.3. Optimization for HDE

**Figure 3:**
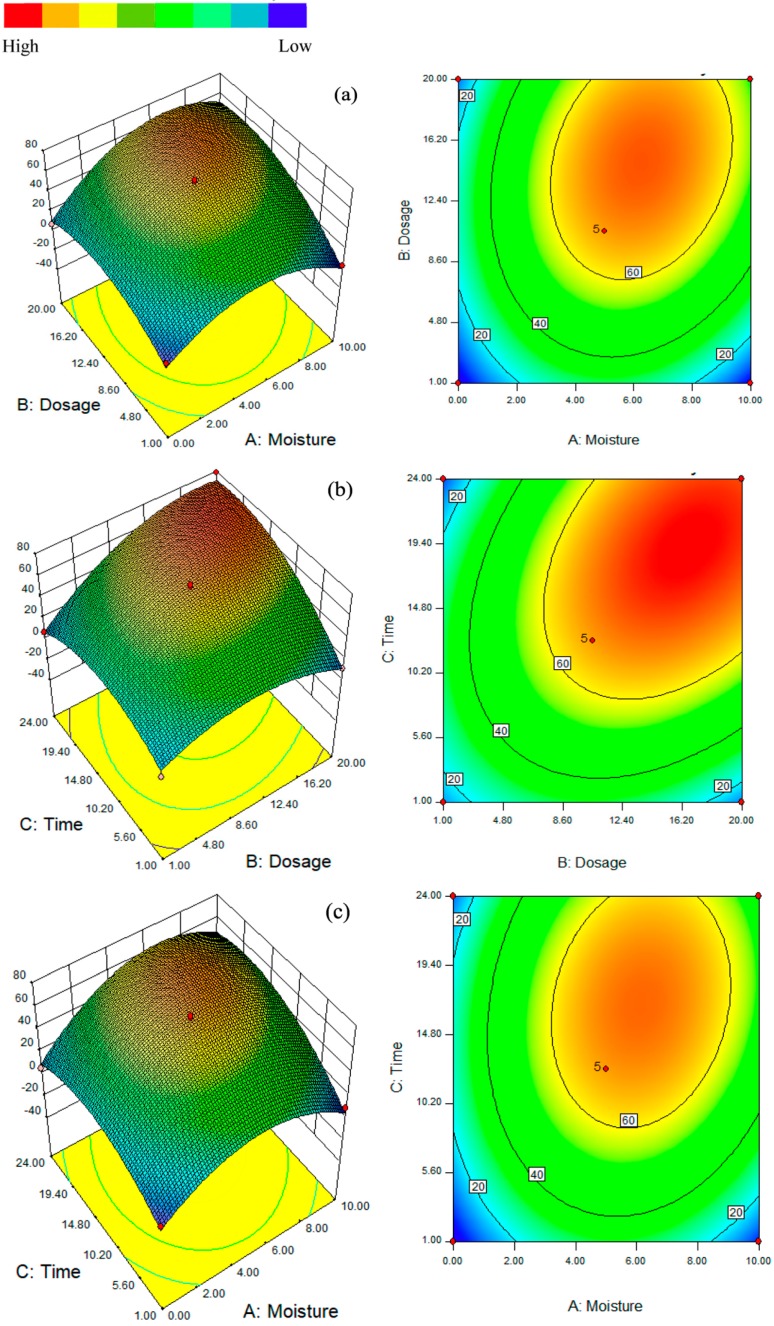
The response surface and contour plots of HDE (%) as the function of (**a**) soil moisture content (%) and n-Al/CaO dosage (%), (**b**) n-Al/CaO dosage (%) and grinding time (h), (**c**) soil moisture content (%) and grinding time (h).

To better evaluate the impacts of the three variables (soil moisture content, n-Al/CaO dosage, and grinding time) on HDE, the predictive quadratic model was presented as three-dimensional response surface plots and two-dimensional contours in Figure 3. The results given in this figure showed the effects of two variables on the HDE while another variable remained constant at its zero level (Table 1). The maximum HDE of 80% was achieved at a soil moisture content of 7.0%, a n-Al/CaO dosage of 17.7%, and a grinding time of 24 h. Compared with the common technologies such as AOPs, nZVI treatment, and bioremediation, the n-Al/CaO’s application in the remediation of HCB-contaminated soils is easier to be operated with higher decomposition efficiency. In an electrokinetic Fenton process, the maximum of 57%–64% (initial concentration 100 mg/kg) HCB in the kaolin was removed after 14 days [33,34,35]. As for nZVI treatment (mixture of 15 mL HCB and 2 g nZVI), the dechlorination efficiency is 60% after 24 h stirring on a rotary shaker (125 r/min). In a novel biochar-plant tandem approach, the degradation efficiency can be 56.11% after a six-month growing period [20].

Figure 3a showed the interaction of the n-Al/CaO dosage and soil moisture content on HDE at the grinding time of 12.5 h. The HDE increased significantly with an increase in the n-Al/CaO dosage, which may be attributed to more contact surface and reactive sites of n-Al/CaO. As for soil moisture, no matter what the n-Al/CaO dosage was, the HDE first increased and then decreased with the increase of soil moisture content. The *p*-value of 0.0119 (AB) indicated that the interactions between the two variables on the HDE were significant. In Figure 3b, the HCB dechlorination efficiency generally increased with an increase in the n-Al/CaO dosage and the grinding time. When the n-Al/CaO dosage was low, the HDE increased slightly with the increase of grinding time. However, the HDE increased sharply when the n-Al/CaO was high. Figure 3c illustrated the effect of soil moisture content and grinding time by fixing the n-Al/CaO dosage, which clearly showed that lack or excess of soil moisture content was adverse to the HCB dechlorination process regardless of the grinding time.

The additional experiments were carried out to verify the consistency of the results calculated from the predictive quadratic model and the experimental results under optimum conditions. The experimental HDE of 80.60% was in close agreement with the estimated consequences by the model.

### 3.4. The Pathways and Mechanism of HCB Dechlorination

**Figure 4:**
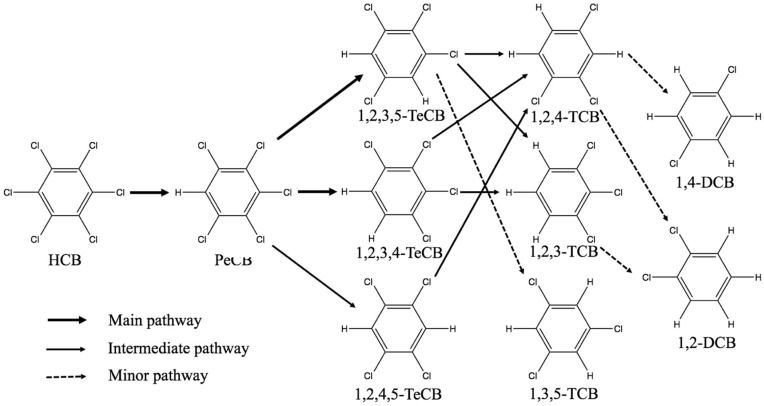
Dechlorination pathways of HCB.

The pathway analysis for HCB dechlorination was illustrated by the case of optimal condition in the RSM model (soil moisture 7.0% and n-Al/CaO dosage 17.7%). The mass balance of chlorobenzenes was shown in Table 4. The reductive HCB dechlorination by successive loss of chloride atoms is confirmed as the main pathway with n-Al/CaO treatment (Figure 4). After 24 h of treatment, the products detected were PeCB, TeCB, trichlorobenzene (TCB) and a small amount of dichlorobenzene (DCB) (less than 2%), which was not in agreement with several dechlorination studies of HCB with zero-valent iron and other reagents [36,37]. As for nanoscale Pd/Fe particles or subcolloidal Ag/Fe particles, 80% of dechlorination intermediates were determined as three TeCBs and 1,2,4- and 1,3,5-TCBs [38,39]. PeCB, three TeCBs, two TCBs (1,2,3- and 1,2,4-), and one DCBs (1,2-DCB) were found by Zhu et al. [40] after nanoscale Cu/Fe particle treatment of HCB. In this study, Figure 4 shows the pathway of HCB dechlorination. PeCB, 1,2,3,5-TeCB, and 1,2,3,4-TeCB accounted for more than 80% of all the dechlorination products, which implied the main pathway. During the initial grinding period of HCB (1 h), the first product detected by GC was PeCB. After 12.5 h of n-Al/CaO treatment, 1,2,3,5-TeCB and 1,2,3,4-TeCB were the main product of PeCB, and 1,2,4,5-TeCB was only present in minor amounts (less than 2%). And after 24 h, 1,2,3-TCB and 1,2,4-TCB were detected (more than 2%), only a trace of 1, 3, 5-TCB, 1, 2-DCB, and 1, 4-DCB were detected. 1,3-DCB and chlorobenzene were not detected. Therefore, the main pathway within 24 h is HCB → PeCB → 1,2,3,4-TeCB and 1,2,4,5-TeCB.

According to the mechanochemical theory, a probable mechanism of the dechlorination process is based on the hydrogen radical substitution reaction [28,41,42]. The mechanism of n-Al/CaO treatment is different with other common technologies such as AOPs and nZVI treatment, which decompose the HCB through the production of hydroxyl radical (·OH) or the oxidative dissolution of the zero-valent Fe [43,44]. We assume that the reaction of n-Al/CaO in the contaminated soil treatment occurs through a stepwise reductive dechlorination process may be given as four steps below (*n* = 6, 5, 4, 3):

(1) Al → Al^3+^ + 3e^−^

(2) e^−^ + H_2_O → OH^−^ + H• or 1/2H_2_

(3) e^−^ + Rh-Cl*_n_* → Cl_*n*−1_-Rh• + Cl^−^

(4) Cl_*n*−1_-Rh• + H• → Cl_*n*−1_-Rh-H

The oxidation-reduction potential of Al^3+^/Al is −1.662 V, which makes the metallic aluminum a reducing agent and an electron donor Equation (1). During simple grinding, electrons can directly interact with water and yield hydrogen radicals Equation (2). Due to the homolytic dissociation of C-Cl bond in a triboplasma state, [28,45] the chlorinated benzenes can also react with electrons Equation (3). After this process, the free radicals combine and yield the hydrodechlorinated reaction product Equation (4).

With the high specific surface area and reaction activity of n-Al/CaO, the HCB was dechlorinated in the presence of a proton donor like water and the chlorine atoms were scavenged by CaO [46,47]. In this process, CaO can be a hydrodechlorinating reagent, a dryer, and a protector of Al [27,28]. Furthermore, the CaO can result in alkaline conditions, which helps catalyze the HCB dechlorination [16].

Soil moisture content plays an important role in the dechlorination process according to Equation (2). The water can act as a proton donor to stimulate the hydrodechlorination process. The low hydrodechlorination efficiency (less than 8%) at the soil moisture content of 0% may prove this. Furthermore, water can react with CaO and generate heat, which increases the hydrodechlorination rate of HCB [38,48]. In this way, the HCB in the contaminated soils was dechlorinated.

### 3.5. The Environmental and Economic Analysis

In the present study, n-Al/CaO could be a potential environmentally-friendly and cost-effective material. Both Al and Ca are common elements in soils, so the addition of n-Al/CaO to soils with a relatively low loading ratio will not greatly change the compositions of the soils, which maintains the soil structure, properties, and function after remediation. Meanwhile, the toxicity of HCB decreases with the successive loss of chlorine atoms during n-Al/CaO treatment, which will greatly lower the environmental risk of the contaminated soils [21]. Also, in the presence of moisture and atmospheric CO_2_, the remaining CaO could form carbonates which do not harm the environment [27]. Regarding the economic efficiency of this remediation technology, even though it is difficult to accurately estimate the cost of using n-Al/CaO to remediate HCB-contaminated soils, this n-Al/CaO proves to be much cheaper than other similar materials, such as n-Ca/CaO. According to cost estimates of n-Ca/CaO treatment, the cost of treating one ton of contaminated soils was reported as $40 [49]. The preparation method, addition dosage, and remediation process of n-Al/CaO are similar with n-Ca/CaO, while the storage conditions of n-Al/CaO are simpler and Al is much cheaper than Ca. Therefore, the cost of treatment for one ton of HCB-contaminated soils should be less than $40. The accurate estimation of the cost for this technique would be practical based on the data obtained through land application of n-Al/CaO in real scenarios.

## 4. Conclusions

The study explored the potential application of n-Al/CaO for the dechlorination of HCB in contaminated soils. The high HCB dechlorination efficiency (up to 80%) seems promising. RSM was employed to optimize three variables (soil moisture, n-Al/CaO dosage, and grinding time) and revealed that the predictive quadratic model is adequate to explain the dechlorination process as the *R*^2^ value was 0.9807. According to the ANOVA results, the predictive model was proved appropriate to describe the correlation between the variables and the responses. The optimum dechlorination conditions were found to be soil moisture content of 7.0%, n-Al/CaO dosage of 17.7%, and a grinding time of 24 h. Under optimized conditions, simple grinding can achieve 80% of HCB dechlorination in the contaminated soils, while the main products are PeCB, 1,2,3,4-TeCB and 1,2,4,5-TeCB. These results suggest that water plays an important role in the dechlorination process. The probable mechanism is based on a hydrogen radical substitution. Overall, n-Al/CaO has the potential to remediate the HCB-contaminated soils in engineering practice under natural moisture and atmosphere conditions.

## Figures and Tables

**Table 1:** Experimental range and level of the independent variables.

Variable	Code	Range and Level		
		−1	0	1
Soil Moisture content (%)	A	0	5	10
n-Al/CaO dosage (%)	B	1	10.5	20
Grinding time (h)	C	1	12.5	24

**Table 2:** The experimental results based on the three-level Box-Behnken design.

Run	*A*	*B*	*C*	HDE (%)
1	5.00	10.50	12.50	69.4
2	5.00	10.50	12.50	67.8
3	5.00	10.50	12.50	65.1
4	5.00	20.00	24.00	78.8
5	0.00	10.50	1.00	1.45
6	5.00	10.50	12.50	61.4
7	10.00	10.50	1.00	8.79
8	5.00	10.50	12.50	68.7
9	5.00	1.00	24.00	7.54
10	10.00	10.50	24.00	44.9
11	10.00	20.00	12.50	48.3
12	0.00	1.00	12.50	3.23
13	5.00	20.00	1.00	11.4
14	10.00	1.00	12.50	4.26
15	5.00	1.00	1.00	6.38
16	0.00	20.00	12.50	7.64
17	0.00	10.50	24.00	5.71

**Table 3:** ANOVA analysis of the model.

Source	Sum of Squares	Df	Mean Square	F Value	*p*-Value (Prob > F)
Model	14383.85	9	1598.21	46.23	<0.0001
A-Moisture	972.85	1	972.85	28.14	0.0011
B-Dosage	1944.70	1	1944.70	56.26	0.0001
C-Time	1483.22	1	1483.22	42.91	0.0003
AB	392.63	1	392.63	11.36	0.0119
AC	253.61	1	253.61	7.34	0.0303
BC	1096.93	1	1096.93	31.73	0.0008
A^2^	3973.55	1	3973.55	114.94	<0.0001
B^2^	1667.83	1	1667.83	48.25	0.0002
C^2^	1777.68	1	1777.68	51.42	0.0002
Residual	241.98	7	34.57		
Lack of Fit	199.08	3	66.36	6.19	0.0553
Pure Error	42.91	4	10.73		
Cor Total	14625.83	16			

**Table 4:** Mass balance of chlorobenzenes *.

Time (h)	0		1		12.5		24	
	μmol/kg	%	μmol/kg	%	μmol/kg	%	μmol/kg	%
HCB	44.50	100	37.24	88.18	13.67	34.81	7.07	19.27
PeCB	-	-	4.49	10.63	19.51	49.68	14.31	39.01
1,2,3,4-TeCB	-	-	0.36	0.85	3.26	8.30	5.67	15.46
1,2,3,5-TeCB	-	-	0.11	0.26	1.95	4.97	3.76	10.25
1,2,4,5-TeCB	-	-	0.03	0.08	0.57	1.45	2.90	7.91
1,2,3-TCB	-	-	-	-	0.21	0.53	0.96	2.62
1,2,4-TCB	-	-	-	-	0.10	0.26	0.91	2.48
1,3,5-TCB	-		-	-	-	-	0.43	1.17
1,2-DCB	-	-	-	-	-	-	0.51	1.39
1,4-DCB	-	-	-	-	-	-	0.16	0.44

* All the measurements were in triplicate and measurement errors were within ±5%.

## References

[1] Wang L., Zhang S., Wang L., Zhang W., Shi X., Lu X., Li X., Li X. (2018). Concentration and risk evaluation of polycyclic aromatic hydrocarbons in urban soil in the typical semi-arid city of Xi’an in Northwest China. Int. J. Environ. Res. Public Health.

[2] Jiang L., Wang Q., Liu H., Yao J. (2015). Influence of degradation behavior of coexisting chlorobenzene congeners pentachlorobenzene, 1,2,4,5-tetrachlorobenzene, and 1,2,4-trichlorobenzene on the anaerobic reductive dechlorination of hexachlorobenzene in dye plant contaminated soil. Water Air Soil Pollut..

[3] Alonso F., Beletskaya I.P., Yus M. (2002). Metal-mediated reductive hydrodehalogenation of organic halides. Chem. Rev..

[4] Bailey R.E. (2001). Global hexachlorobenzene emissions. Chemosphere.

[5] Khan S., Priyamvada S., Khan S.A., Khan W., Yusufi A. (2018). Studies on hexachlorobenzene (HCB) induced toxicity and oxidative damage in the kidney and other rat tissues. Int. J. Toxicol..

[6] Porpora M.G., Lucchini R., Abballe A., Ingelido A.M., Valentini S., Fuggetta E. (2013). Placental transfer of persistent organic pollutants: A preliminary study on mother-newborn pairs. Int. J. Environ. Res. Public Health.

[7] Zhang K., Huang J., Wang H., Liu K., Yu G., Deng S., Wang B. (2014). Mechanochemical degradation of hexabromocyclododecane and approaches for the remediation of its contaminated soil. Chemosphere.

[8] Vrana B., Paschke A., Popp P., Schüürmann G. (2004). Use of semipermeable membrane devices (SPMDs). Determination of bioavailable, organic, waterborne contaminants in the industrial region of Bitterfeld, Saxony-Anhalt, Germany. Aquat. Toxicol..

[9] Brown P. (2009). Toxic waste in our midst: Towards an interdisciplinary analysis. J. Environ. Manag..

[10] Nascimento N.R.D., Nicola S.M.C., Rezende M.O.O., Oliveira T.A., Öberg G. (2004). Pollution by hexachlorobenzene and pentachlorophenol in the coastal plain of São Paulo state, Brazil. Geoderma.

[11] Barber J.L., Sweetman A.J., Van W.D., Jones K.C. (2005). Hexachlorobenzene in the global environment: Emissions, levels, distribution, trends and processes. Sci. Total. Environ..

[12] Wang G., Lu Y., Han J., Luo W., Shi Y., Wang T., Sun Y. (2010). Hexachlorobenzene sources, levels and human exposure in the environment of China. Environ. Int..

[13] USEPA (2013). Superfund Remedy Report (14th Edition). https://www.epa.gov/superfund.

[14] Li T., Yuan S., Wan J., Lu X. (2010). Hydroxypropyl-β-cyclodextrin enhanced electrokinetic remediation of sediment contaminated with HCB and heavy metals. J. Hazard. Mater..

[15] Wan J., Li Z., Lu X., Yuan S. (2010). Remediation of a hexachlorobenzene-contaminated soil by surfactant-enhanced electrokinetics coupled with microscale Pd/Fe PRB. J. Hazard. Mater..

[16] Huang H., Jiang J., Xiao Y., Chen X., Liu S. (2014). A novel and efficient method for dechlorination of hexachlorobenzene using a sodium carbonate/glycerol system. Chem. Eng. J..

[17] Adusei-Gyamfi J., Acha V. (2016). Carriers for nano zerovalent iron (nZVI): Synthesis, application and efficiency. RSC Adv..

[18] Zhao L., Zhu T., Hou H., Qin X., Li F., Terada A., Hosomi M. (2015). Removal of PCBs and HCB from contaminated solids using a novel successive self-propagated sintering process. Environ. Sci. Pollut. Res..

[19] Sievers M., Wilderer P. (2011). Advanced oxidation processes. Treatise on Water Science.

[20] Song Y., Li Y., Zhang W., Wang F., Bian Y., Boughner L.A., Jiang X. (2016). Novel Biochar-Plant Tandem Approach for Remediating Hexachlorobenzene Contaminated Soils: Proof-of-Concept and New Insight into the Rhizosphere. J. Agric. Food. Chem..

[21] Figueroa I.D.C., Simmons M.S. (2010). Structure-activity relationships of chlorobenzenes using DNA measurement as a toxicity parameter in algae. Environ. Toxicol. Chem..

[22] Zhang W., Wang H., Jun H., Yu M., Wang F., Zhou L., Yu G. (2014). Acceleration and mechanistic studies of the mechanochemical dechlorination of HCB with iron powder and quartz sand. Chem. Eng. J..

[23] Mulas G.E.A. (1997). The Mechanochemical Self-Propagating Reaction between Hexachlorobenzene and Calcium Hydride. J. Solid State Chem..

[24] Lu S., Huang J., Peng Z., Li X., Yan J. (2012). Ball milling 2,4,6-trichlorophenol with calcium oxide: Dechlorination experiment and mechanism considerations. Chem. Eng. J..

[25] Ren Y., Kang S., Zhu J. (2015). Mechanochemical degradation of hexachlorobenzene using Mg/Al_2_O_3_ as additive. J. Mater. Cycles Waste Manag..

[26] Li Y., Liu Q., Li W., Lu Y., Meng H., Li C. (2017). Efficient destruction of hexachlorobenzene by calcium carbide through mechanochemical reaction in a planetary ball mill. Chemosphere.

[27] Mallampati S.R., Mitoma Y., Okuda T., Sakita S., Simion C. (2014). Simultaneous decontamination of cross-polluted soils with heavy metals and PCBs using a nano-metallic Ca/CaO dispersion mixture. Environ. Sci. Pollut. Res..

[28] Mitoma Y., Simion A.M., Mallampati S.R., Miyata H., Kakeda M., Simion C. (2015). Hydrodechlorination of PCDD/PCDF/PCB contaminants by simple grinding of contaminated soils with a nano-size calcium reagent. Environ. Prog. Sustain. Energy.

[29] Yuan S., Tian M., Lu X. (2006). Microwave remediation of soil contaminated with hexachlorobenzene. J. Hazard. Mater..

[30] Wan J., Yuan S., Mak K., Chen J., Li T., Lin L., Lu X. (2009). Enhanced washing of HCB contaminated soils by methyl-β-cyclodextrin combined with ethanol. Chemosphere.

[31] Nie X., Liu J., Yue D., Zeng X., Nie Y. (2013). Dechlorination of hexachlorobenzene using lead/iron bimetallic particles. Chemosphere.

[32] Jensen W.A. (2017). Response Surface Methodology: Process and Product Optimization Using Designed Experiments. J. Qual. Technol..

[33] Oonnittan A., Shrestha R.A., Sillanpää M. (2009). Removal of hexachlorobenzene from soil by electrokinetically enhanced chemical oxidation. J. Hazard. Mater..

[34] Oonnittan A., Isosaari P., Sillanpää M. (2010). Oxidant availability in soil and its effect on HCB removal during electrokinetic Fenton process. Sep. Purif. Technol..

[35] Oonnittan A., Shrestha R.A., Sillanpää M. (2008). Remediation of hexachlorobenzene in soil by enhanced electrokinetic Fenton process. J. Environ. Sci. Health A.

[36] Roy G., de Donato P., Görner T., Barres O. (2003). Study of tropaeolin degradation by iron—Proposition of a reaction mechanism. Water Res..

[37] Shih Y.-H., Hsu C.-Y., Su Y.-F. (2011). Reduction of hexachlorobenzene by nanoscale zero-valent iron: Kinetics, pH effect, and degradation mechanism. Sep. Purif. Technol..

[38] Shih Y.-H., Chen Y.-C., Chen M.-Y., Tai Y.-T., Tso C.-P. (2009). Dechlorination of hexachlorobenzene by using nanoscale Fe and nanoscale Pd/Fe bimetallic particles. Colloid Surf. A.

[39] Xu Y., Zhang W.-X. (2000). Subcolloidal Fe/Ag particles for reductive dehalogenation of chlorinated benzenes. Ind. Eng. Chem. Res..

[40] Zhu N., Luan H., Yuan S., Chen J., Wu X., Wang L. (2010). Effective dechlorination of HCB by nanoscale Cu/Fe particles. J. Hazard. Mater..

[41] Nomura Y., Nakai S., Hosomi M. (2005). Elucidation of degradation mechanism of dioxins during mechanochemical treatment. Environ. Sci. Technol..

[42] Nomura Y., Fujiwara K., Terada A., Nakai S., Hosomi M. (2012). Mechanchemical degradation of γ-hexachlorocyclohexane by a planetary ball mill in the presence of CaO. Chemosphere.

[43] Wang N., Zheng T., Zhang G., Wang P. (2016). A review on Fenton-like processes for organic wastewater treatment. J. Environ. Chem. Eng..

[44] Matheson L.J., Tratnyek P.G. (1994). Reductive dehalogenation of chlorinated methanes by iron metal. Environ. Sci. Technol..

[45] Heineke G. (1984). Tribochemistry.

[46] Yan J.H., Peng Z., Lu S.Y., Li X.D., Ni M.J., Cen K.F., Dai H.F. (2007). Degradation of PCDD/Fs by mechanochemical treatment of fly ash from medical waste incineration. J. Hazard. Mater..

[47] Mitoma Y., Egashira N., Simion C. (2009). Highly effective degradation of polychlorinated biphenyls in soil mediated by a Ca/Rh bicatalytic system. Chemosphere.

[48] Mitoma Y., Mallampati S.R., Miyata H., Kakeda M. (2012). Decomposition of polychlorinated biphenyls in soil with a dispersion mixture of metallic calcium and calcium oxide. Arch. Environ. Contam. Toxicol..

[49] Mallampati S.R., Mitoma Y., Okuda T., Sakita S., Kakeda M. (2012). Total immobilization of soil heavy metals with nano-Fe/Ca/CaO dispersion mixtures. Environ. Chem. Lett..

